# Recovery and evolutionary analysis of complete integron gene cassette arrays from *Vibrio*

**DOI:** 10.1186/1471-2148-6-3

**Published:** 2006-01-18

**Authors:** Yan Boucher, Camilla L Nesbø, Michael J Joss, Andrew Robinson, Bridget C Mabbutt, Michael R Gillings, W Ford Doolittle, HW Stokes

**Affiliations:** 1 Department of Chemistry and Biomolecular Sciences, Macquarie University, Sydney, NSW 2109, Australia; 2 Genome Atlantic, Dalhousie University, Halifax, Nova Scotia, 5859 University Avenue, B3H 4H7, Canada; 3 Department of Biological Sciences, Macquarie University, Sydney, NSW 2109, Australia

## Abstract

**Background:**

Integrons are genetic elements capable of the acquisition, rearrangement and expression of genes contained in gene cassettes. Gene cassettes generally consist of a promoterless gene associated with a recombination site known as a 59-base element (59-be). Multiple insertion events can lead to the assembly of large integron-associated cassette arrays. The most striking examples are found in *Vibrio*, where such cassette arrays are widespread and can range from 30 kb to 150 kb. Besides those found in completely sequenced genomes, no such array has yet been recovered in its entirety. We describe an approach to systematically isolate, sequence and annotate large integron gene cassette arrays from bacterial strains.

**Results:**

The complete *Vibrio sp*. DAT722 integron cassette array was determined through the streamlined approach described here. To place it in an evolutionary context, we compare the DAT722 array to known vibrio arrays and performed phylogenetic analyses for all of its components (integrase, 59-be sites, gene cassette encoded genes). It differs extensively in terms of genomic context as well as gene cassette content and organization. The phylogenetic tree of the 59-be sites collectively found in the *Vibrio *gene cassette pool suggests frequent transfer of cassettes within and between *Vibrio *species, with slower transfer rates between more phylogenetically distant relatives. We also identify multiple cases where non-integron chromosomal genes seem to have been assembled into gene cassettes and others where cassettes have been inserted into chromosomal locations outside integrons.

**Conclusion:**

Our systematic approach greatly facilitates the isolation and annotation of large integrons gene cassette arrays. Comparative analysis of the *Vibrio sp*. DAT722 integron obtained through this approach to those found in other vibrios confirms the role of this genetic element in promoting lateral gene transfer and suggests a high rate of gene gain/loss relative to most other loci on vibrio chromosomes. We identify a relationship between the phylogenetic distance separating two species and the rate at which they exchange gene cassettes, interactions between the non-mobile portion of bacterial genomes and the vibrio gene cassette pool as well as intragenomic translocation events of integrons in vibrios.

## Background

The integron/gene cassette system is now known to be widely distributed amongst the Bacteria and is a major contributor to the dispersal of genes by Lateral Gene Transfer (LGT). The system can facilitate LGT by virtue of the fact that the integron encodes a site-specific recombination (SSR) system [1,2]. The targets for SSR are gene cassettes [3]. These are independently mobilizable units of DNA, usually composed of a single gene bound by a recombination site, designated a 59-base element (59-be) or alternatively *attC*. The DNA integrase catalysing SSR, IntI, is encoded by the integron. This integrase recognises two families of recombination sites. The first of these is the cassette associated 59-be [4] and the second is a site contained within the integron designated *attI*. IntI can catalyze a reversible reaction by which gene cassettes can be inserted into, or excised from, an integron via recombination between the *attI *site and a 59-be or between two 59-be sites [5,6]. Multiple recombination events are common. As a consequence, integrons are normally associated with arrays comprising multiple cassettes, which in some species of *Vibrio *can number well in excess of a hundred [7]. Gene cassettes do not normally include a promoter. Instead, where it has been examined, transcription of cassette genes is driven by a promoter (P_c_) located in the integron itself [1]. Thus, the integron and gene cassettes comprise both a gene capture and gene expression system.

**Figure 1:**
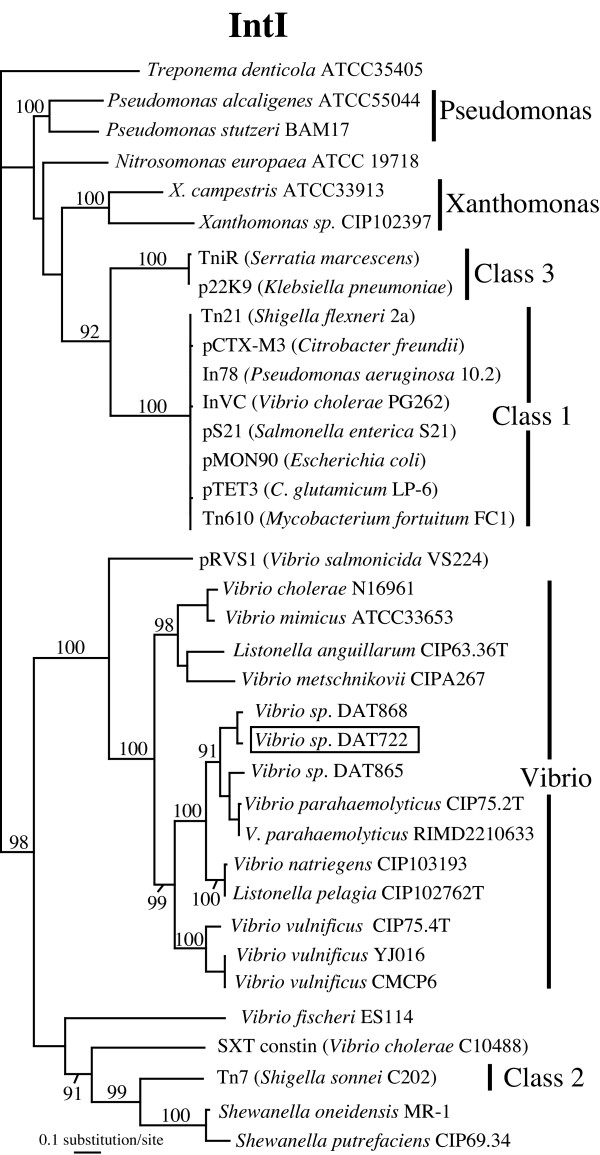
**Best maximum likelihood phylogeny of representative integron integrases (IntI). **For integrons carried on a mobile element such as a plasmid or transposon, the taxon name represents this genetic element and the host name is in parentheses. Some integron integrases can be attributed to a specific integron class, as indicated by the name of the clade to which they belong. Support values displayed above the nodes represent the consensus of the best maximum likelihood distance trees of 100 bootstrap pseudo-replicates of the dataset (only values >80% are displayed). The sequences of the intI genes from Vibrio sp. DAT865, DAT868 and DAT722 strains were obtained in this study.

Integrons were first identified in the context of multi-drug resistance. In this context, pathogenic bacteria are often resistant to many antibiotics as a consequence of possessing an integron that has captured several gene cassettes containing resistance genes [2]. These integrons recovered from clinical environments are often embedded in other types of mobile elements such as plasmids and transposons [8]. It is now clear however that integrons are also a common feature of the chromosomes of various bacteria, being found in the gamma-proteobacteria (vibrios, xanthomonads, pseudomonads, *Shewanella*), beta-proteobacteria (*Nitrosomonas europea*) and spirochaetes (*Treponema denticola*) (Figure 1). Another notable feature of chromosomal integrons is the wide diversity of functions encoded by the genes found in their gene cassettes [9]. This, coupled with the observation that cassette arrays can be quite large, clearly hints at a system that can greatly impact on the adaptive potential of bacteria [10]. Given that cassette associated genes are part of the gene pool that is LGT-associated and mobilizable, they also represent a community resource that can be shared between individuals [11].

Bacteria of the *Vibrio *genus are rapidly becoming a model system for the study of the chromosomal integron/gene cassette system [12-14]. As noted above, their cassette arrays are characteristically large, encompassing several percent of genomic coding capacity in many cases. Representatives of this genus display such a high level of variation in terms genome size and content that "species" units can rarely be identified by the sequencing of one or a few genetic markers [15]. Many complete integron arrays have now been assembled from various species of *Vibrio *as a result of whole genome sequencing initiatives. These include *V. cholerae, V. parahaemolyticus, V. vulnificus *(two strains) and *V. fischeri *[16-19]. These collective sequencing efforts have helped to highlight the enormous amount of novel genetic diversity contained within integron arrays. However, it is also becoming clear that whole genome sequencing is a relatively blunt instrument for assessing gene cassette diversity. This is because, although individual strains of *Vibrio *can contain large arrays, the total number of cassettes harboured by any single individual is very small compared to the overall size of this community resource [11]. As an alternative approach to recovering gene cassettes, the cassette PCR technique has been developed [20]. This method selectively amplifies gene cassettes to the exclusion of other genomic sequences and can be applied to both metagenomic (environmental) DNA and to the DNA of defined strains. However, cassette PCR only recovers cassettes in isolation (i.e. outside their genetic context) and cannot aid in the assembly of contiguous arrays. Also, although gene cassette PCR is selective, there is always the possibility for some false positives (non-cassette DNA fragments being amplified) [20]. When a gene cassette is found within an array, there is no doubt about its nature.

**Figure 2:**
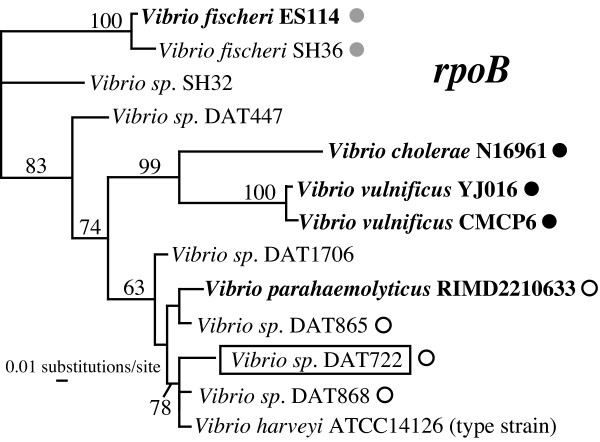
**Best maximum likelihood phylogeny of *Vibrio *species based on the RNA polymerase B gene (*rpoB*). **The strain which had its entire integron gene cassette array sequenced in this study is boxed. Organisms that had their complete genome sequenced are in bold (the sequence of the *rpoB *genes of all other strains were generated in this study). The gene immediately upstream of the integron, when known, is indicated next to the taxon name: white dot (hypothetical protein VC1310), black dot (ribosomal protein RplT) or grey dot (hypothetical protein VCA0034). Support values displayed above the nodes represent the consensus of the best maximum likelihood trees of 100 bootstrap pseudo-replicates of the dataset (only values >50% are displayed).

To more rapidly access and analyse the mobile gene cassette pool, we adopted a genomics approach that allows us to specifically isolate DNA fragments containing large integron gene cassette arrays and exclude the vast majority of the genome that is common to most members of a species. No such large cassette array has previously been isolated and fully sequenced without completely sequencing the genome of its host. Using our streamlined approach, we isolate and sequence the integron from a close relative of the widespread marine bacterium *Vibrio harveyi*, a well-known member of the core vibrio group to which *V. parahaemolyticus *also belongs (Figure 2). To place it in an evolutionary context, we compare it to known vibrio integron arrays and perform a phylogenetic analysis of all of its components (integrase, 59-be sites, gene cassette encoded genes). This reveals strong interaction between vibrio integrons through LGT, high variability of array contents and wide functional diversity of gene cassettes. These analyses also yield insights on the processes of gene cassette recruitment from non-mobile genes and the possibility of cassette de-recruitment. Differences in the genomic context of integrons from various vibrios suggest several events of intragenomic translocation for these genetic elements.

## Results and discussion

### Characterization of the *Vibrio sp*. DAT722 cassette array

The method used to isolate a complete vibrio gene cassette array takes advantage of the fact that cassettes are linked, having been assembled at a single locus, the integron *attI *site. By making genomic libraries and screening individual clones by cassette PCR or with PCR primers targeting the *intI *gene, we can recover large inserts that comprise exclusively, or in large part, of contiguous arrays of mobile gene cassettes. Here we demonstrate this principle on an environmental strain of a *Vibrio sp*. from an aquaculture facility in Darwin, Australia. We show that using a fosmid vector (insert size between 30–40 kb) to construct a genomic library (480 clones), we can recover the whole 82 kb integron array in four PCR screening rounds, the first of which identifies a clone bearing the *intI*. A structured query language (SQL) script was developed to facilitate annotation of integron arrays, made difficult by the presence of non-coding elements such as 59-be sites. The script performs a search for short DNA motifs on a DNA segment and detects intervening ORFs, outputting annotation in GenBank format.

**Figure 3:**
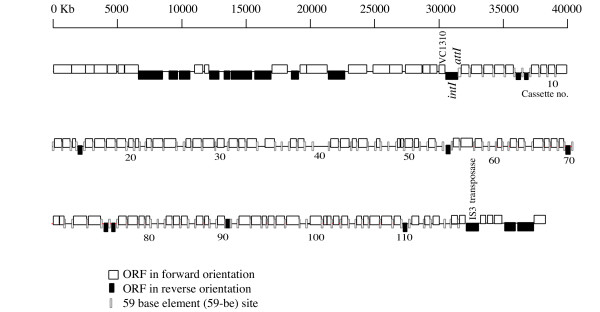
Diagram of the *Vibrio sp*. DAT722 integron gene cassette array

The gene cassette array of *Vibrio sp*. isolate DAT722 has an average G+C content of 40.7%, compared to 44.8% for the flanking DNA regions. A G+C content lower than the genomic average of the host is typical of vibrio integrons, and differences ranging from 2.8 to 5.7% can be found in completely sequenced vibrio genomes harbouring an integron (data not shown). The DAT722 array contains 116 gene cassettes (Figure 3), falling between the shortest vibrio array of 36 cassettes (*V. fischeri*) and the longest array of 219 cassettes (*V. vulnificus *CMCP6) (Table 1). However, DAT722 contains a high diversity of cassettes, comprising 94 different cassette types out of a total of 116 cassettes. A cassette type represents either a non-paralogous cassette or a group of paralogous cassettes, the latter being defined as a group of cassette that have reciprocal BLASTP or BLASTN matches with an e-value of 1E-10 or less). This number of different cassette types is only equalled or surpassed by much larger arrays (*V. cholerae *with 95 cassette types of a total 173 cassettes and *V. vulnificus *CMCP6 with 141 cassette types of a total 219 cassettes). Consistent with this cassette diversity, the DAT722 array also encodes a wide variety of functions including DNA modification and primary metabolic enzymes (Table 2). Also typical of gene cassette arrays, there is a high proportion of cassettes either with no detectable homolog (37 cassettes) or with sequence matches only to hypothetical proteins (40 cassettes). Only a minority of cassettes encodes a protein to which a putative function can be assigned: 8 cassettes can be assigned a specific function with reasonable certainty and another 12 a broad function (for example matching a general super-family of proteins such as acetyltransferases).

**Table 1:** Characteristics of *Vibrio *integrons

	VvuCM^a^	VvuYJ	Vch	Vpa	Vfi	VspDAT
length (kb)	151	138	125	47	29	82
% of host genome	2.9%	2.6%	3.1%	0.9%	0.7%	n.d.^b^
no. cassettes	219	187	173	69	36	116
non-coding	107	57	11	8	7	25
coding	112	130	162	61	29	91
no. cassette types	141	88	95	59	31	94
paralogous groups	18	28	44	7	2	9
singlets	123	60	51	52	29	85

^a ^VvuCM (*Vibrio vulnificus *CMCP6); VvuYJ (*Vibrio vulnificus *YJ016); Vch (*Vibrio cholerae *N16961); Vpa (*Vibrio parahaemolyticus *RIMD2210633); Vfi (*Vibrio fischeri *ES114); VspDAT (*Vibrio sp*. DAT722) ^b^n.d. (not determined)

**Table 2:** Identifiable functions of the *Vibrio sp*. DAT722 gene cassette encoded proteins

Cassette no.	Putative function	Source of best hit^a^	E-value	Identity (%)
	*DNA modification*			
11	DNA topoisomerase I	*Shewanella amazonensis*	1E-96	68
57	NUDIX hydrolase	*Deinococcus radiodurans*	1E-29	46
59	DNA topology modulation protein	*Bacillus cereus*	5E-45	51
	*Primary metabolism*			
5	4-methyl-5(B-hydroxyethyl)-thiazole monophosphate biosynthesis	*Bacillus anthracis*	3E-15	29
31	beta-phosphoglucomutase	*Vibrio vulnificus*	4E-86	70
75	selenocysteine lyase	*Anabaena variabilis*	4E-44	55
78	maltose O-acetyltransferase	*Methanosarcina acetivorans*	9E-41	47
	*Other*			
2	lysogenic conversion protein	Bacteriophage P2-EC5	5E-41	33
20	antibiotic biosynthesis monooxygenase	*Ralstonia eutropha*	3E-34	64
24	acetyltransferase	*Streptomyces avermitilis*	6E-27	33
39	acetyltransferase	*Vibrio parahaemolyticus*	9E-86	99
54	acetyltransferase	*Legionella pneumophila*	1E-17	31
84	acetyltransferase	*Paracoccus denitrificans*	3E-26	39
88 & 91	acetyltransferase	*Vibrio vulnificus*	3E-67	66
103	acetyltransferase	*Vibrio fischeri*	4E-61	76
69	histone acetyltransferase	*Vibrio vulnificus*	1E-16	31
74	haemagglutinin associated protein	*Vibrio cholerae*	1E-116	88
97	cold shock domain	*Vibrio vulnificus*	5E-71	77
102	toxin-antitoxin plasmid stability system	*Vibrio vulnificus*	4E-48	94

^a^BLASTP hits from non-redundant database as of July 2005

Interestingly, *Vibrio *gene cassette arrays vary enormously in the proportions of their contents which is composed of non-coding cassettes. Cassettes are defined as "non-coding" when they have no BLASTX matches and they do not contain ORFs longer than 150 bp. Given this definition, the proportion of the array made-up of non-coding cassettes can vary from 6% (*V. cholerae*) to 49% (*V. vulnificus *CMCP6) and does not appear to be correlated with array size. There seems to be some correlation, however, between array size and content in paralogous (whether coding or non-coding) cassettes, as all large arrays (the two *V. vulnificus *strains and *V. cholerae*) have in excess of 44% of cassettes repeated at least twice in the array while smaller arrays all have around 25% of paralogs (Table 1).

One remarkable feature of non-coding cassettes is that they either have very close homolog(s) in other vibrios (>80% nucleotide identity) or no homologs at all. In the *Vibrio sp*. DAT722 array, of 25 non-coding cassettes, 8 have no significant BLASTN hits and 17 are closely related to non-coding cassettes from other vibrios. None of these cassettes seem to represent pseudo-genes, as they do not display any matches to public databases using amino acid translations of all possible reading frames as query (BLASTX). Furthermore, all cassettes defined as non-coding that do have BLASTN matches in other vibrios are similar to these homologs across their whole length. Non-coding cassettes also tend to be paralogous in all vibrio integrons. Although there are only 6 copies of the most prevalent non-coding cassette in the *Vibrio sp*. DAT722 array, there is a highly paralogous family of non-coding cassettes which has 45 representatives in *V. vulnificus *CMCP6 and 34 in *V. vulnificus *YJ016.

Coding cassettes are also frequently paralogous (i.e. have reciprocal BLASTP or BLASTN matches with an e-value of 1E-10 or less). For example, they account for 19 out of the 31 paralogous cassettes in the DAT722 array. The *V. cholerae *array displays the most paralogy among coding cassettes, boasting 115 proteins with some degree of paralogy. However, only two of its paralogous groups have more than 4 members, even when a generous BLASTP similarity cut-off is used to determine paralogy (e-value 1E-10). Coding cassettes differ most significantly from non-coding cassettes by having homologs both inside and outside of the vibrios. Out of 91 coding cassettes in the *Vibrio sp*. DAT722 array, 29 have no BLASTP or BLASTX hit (e-value cutoff 1E-10), 34 have a best match to other vibrios and 28 have a best match outside vibrios.

### Variability of gene cassette array composition

Comparison of cassette content between vibrio strains/species arrays reveals important variability. The number of cassettes shared between the arrays of two different species of *Vibrio*, counting repeated paralogous cassettes only once, varies between 1 and 14 (arrays contain anywhere from 31 to 141 cassette types) (Table 3). The two *V. vulnificus strains*, respectively containing 88 and 141 cassette types, only have 34 cassettes in common. This represents a 30–39% shared content (based on the two different array sizes), which is much lower than the 78–85% shared gene content of their whole genomes when compared with the same homology criteria (BLAST cut-off of 1E-10). The difference in shared gene content between arrays and genomes is even more pronounced when comparing different species. For example, *V. vulnificus *CMCP6 shares 60–72% of its genomic genes with *V. cholerae *but only 10–15% of its array genes.

**Table 3:** Gene cassettes^a ^shared between the integrons of *Vibrio *species

	VvuCM^b^	VvuYJ	Vch	Vpa	Vfi	VspDAT
VvuCM	141	34	14	8	2	12
VvuYJ	-	88	11	10	1	10
Vch	-	-	95	10	4	6
Vpa	-	-	-	59	2	9
Vfi	-	-	-	-	31	4
VspDAT	-	-	-	-	-	94

^a^Paralogous cassettes are only counted once, the middle diagonal displays the number of different cassette types found in each strain

^b^VvuCM (*Vibrio vulnificus *CMCP6); VvuYJ (*Vibrio vulnificus *YJ016); Vch (*Vibrio cholerae *N16961); Vpa (*Vibrio parahaemolyticus *RIMD2210633); Vfi (*Vibrio fischeri *ES114); VspDAT (*Vibrio sp*. DAT722)

### Origins and fates of gene cassettes

There are multiple examples of cassette-encoded vibrio genes only found in a single vibrio strain but showing strong similarity to chromosomal genes from other types of bacteria. Two striking examples are the proteins coded by cassettes 11 and 75 from the DAT722 array. Cassette 11 encodes a putative DNA topoisomerase that has 68% amino acid identity to a similarly annotated protein from *Shewanella amazonensis *(Table 2). The cassette 11 protein also shares 40% identity with proteins coded by uncultured marine alpha-proteobacteria from the SAR116 cluster. The protein coded by cassette 75 shares 55% identity (e-value 4E-44) with selenocysteine lyase from the cyanobacteria *Anabaena variabilis *yet does not display homology to any other protein in current databases. Another notable case is the hypothetical protein encoded by cassette 112, whose only homologs are found in two *Salmonella *and two *Bordetella *species, with which it shares ~50% amino acid identity. It is not possible to perform meaningful phylogenetic analyses of these genes to get a more complete picture of their evolutionary history, as there are not enough known homologs. However, the high degree of similarity between these cassettes and their non-cassette homologs, combined with the lack of any vibrio homologs, suggest that they have been recruited from chromosomal genes of other types of bacteria.

**Figure 4:**
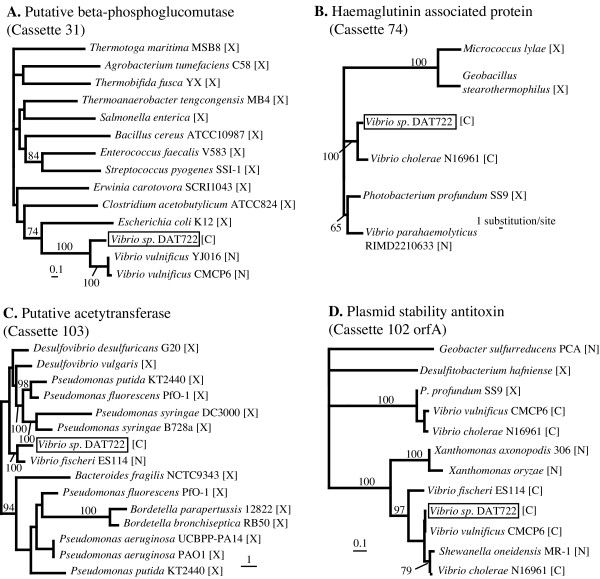
**Best maximum likelihood phylogenies of some *Vibrio sp*. DAT722 gene cassette encoded proteins. **The letter in brackets following the taxon name indicates the genetic context of the gene coding for the protein: C (found in a gene cassette), N (host organism has an integron, but this gene is not found in a gene cassette), X (host organism does not have an integron). Support values displayed above the nodes represent the consensus of the best maximum likelihood distance trees of 100 bootstrap pseudo-replicates of the dataset (only values >50% are displayed).

*Vibrio *cassette-encoded ORFs are also found to have non-cassette ORFs homologs in other vibrio strains. The three most striking cases from the DAT722 array are cassettes 31, 74 and 103. Cassette 31 codes for a putative beta-phosphoglucomutase that has 70% amino acid identity with a non-cassette ORF occuring in both *V. vulnificus *genomes, as well as more distant homologs in a variety of other bacteria (Figure 4A). Cassette 74 (Figure 4B) codes for a haemagglutinin associated protein and shares 88% amino acid identity with a *V. cholerae *cassette-encoded homolog. This cassette product also has 75% identity with a non-cassette protein found in *V. parahaemolyticus *and *Photobacterium profundum*. Cassette 103 (Figure 4C) encodes a putative acetyltransferase that shares 76% identity with a non-cassette ORF in *V. fischeri*, and is additionally found in many other bacteria. No traces of 59-be site can be found next to the non-cassette homologs, and it is unclear wether these vibrio non-cassette ORFs closely related to cassette-encoded ORFs represent cassette generation events or non-specific cassette insertion events. The latter scenario of cassette insertion outside an integron context has been previously documented for gene cassettes generally associated with class 1 integrons [3]. The nature of this secondary site insertion appears to generate a non-functional 59-be thereby preventing subsequent mobilization [3].

There is one clear-cut example of cassette insertion outside an integron emerging from the analysis of the DAT722 array. Cassette 102 encodes two short proteins together forming a toxin-antitoxin stability system widespread in vibrio integrons [18] (a phylogenetic tree of the antoxin protein, which is highly similar to the tree of the toxin protein, is displayed in Figure 4D). The amino acid sequence of the antitoxin protein from DAT722 is 92% identical to its non-cassette homolog in *Shewanella oneidensis *and but only 78% identical to its relative encoded by a cassette in the sequenced *V. fischeri *array. This DAT722 cassette-encoded protein also shares significant similarity to a *Xanthomonas *non-cassette homolog (62% identity). It seems likely in this case that a vibrio gene cassette has been inserted non-specifically in the *S. oneidensis *genome, since the *Shewanella *homolog clusters strongly inside a clade of vibrio cassette-encoded homologs. Although *Shewanella oneidensis *is known to possess a functional integron, it only harbours 3 cassettes and the vibrio-like antitoxin gene is found at a different chromosomal location (Figure 1).

Non-coding cassettes differ from those that are coding in terms of distribution, as no homologs outside the vibrios can be found for these cassettes. Instead, closely related homologs in other vibrio species and frequent paralogs in the same array are found for most non-coding cassettes. For example, non-coding cassette 26 is 84% and 88% identical to paralogous cassettes 46 and 48 at the nucleotide level and shares 89% identity with homologous non-coding *V. parahaemolyticus *and *V. vulnificus *cassettes. This high level of sequence conservation from one species to another suggests that non-coding cassettes are under purifying selection. The degree of sequence conservation of paralogous non-coding cassettes could also be enhanced by a process of gene conversion, where recombination between paralogs leads to a more homogeneous set of gene sequences [21]. This sequence conservation, linked with their limited distribution and frequent paralogy leads to two possibilities: 1) They could be selfish elements associated with vibrio integrons; 2) They have a function beneficial to the integron (contain promoters that regulate neighboring gene cassette encoded gene expression or can be transcribed as RNA molecules that play a role in integron regulation). In either case, they most likely have originated within the vibrios and spread to most representatives of this group harbouring integrons.

### Translocation of integron gene cassette arrays

The genomic context of an integron and its associated cassette array can provide important information on its history. The context of the *Vibrio sp*. DAT722 cassette array differs significantly from the context of its closest sequenced relative, the *V. parahaemolyticus *array. Although the same 16 genes (in an identical order) are found 5' of the integrase gene in both arrays, the context suddenly changes past this conserved block. None of the next 15 genes upstream of this block in DAT722 has a homolog in a nearby location in *V. parahaemolyticus*, some of them are found 0.5 mb away on the large *V. parahaemolyticus *chromosome and two of them match genes found some 35 kb after the end of the array. Similarly, genes found after the end of these two arrays are completely different, with the exception of homologous transposase genes (IS3 family) being found near the last cassette of both arrays. As suggested by Rowe-Magnus *et al*. [12], the fact that the core vibrio group (which includes *V. parahaemolyticus *and *Vibrio sp*. DAT722) integron integrases are flanked by a different gene than their *V. cholerae *and *V. vulnificus *homologs most likely represents an integron translocation event in the ancestor of the former group (Figure 2). It therefore seems that an integron was inserted at a given location (next to the VC1310 gene) in the ancestor of the *V. harveyi */ *V. parahaemolyticus *core vibrio group, and that the array might have then been moved by a transposition or genome rearrangement event. However, since we do not know the degree of synteny (conservation of gene order) between the *V. parahaemolyticus *and *Vibrio sp*. DAT 722 genomes, the possibility that the genome of either of these strains was rearranged on both side of its integron cannot be excluded.

Translocation events like the potential integron movement in the *V. parahaemolyticus *and/or *Vibrio sp*. DAT722 lineage(s) seem likely, as the *V. cholerae *integron has certainly been moved from its original position on the large chromosome, being the only *Vibrio *integron found on the smaller of the two chromosomes typically found in this genus [18]. As is the case for the arrays of *Vibrio sp*. DAT722 and *V. parahaemolyticus*, the block of genes flanking the integron integrase gene is conserved between *V. cholerae *and *V. vulnificus *(limited to 4 genes rather than 16 in this case, including the ribosomal protein gene directly flanking the integrase gene, *rplT*), with entirely different contexts beyond this block. This block of genes would have accompanied the integron when it was translocated from one *V. cholerae *chromosome to the other. Transposases could have been involved, as the *V. cholerae *integron (including the 4 genes block linked to it) is flanked by genes coding for such proteins on both sides.

Those translocation events do not seem to occur solely within and between chromosomes. *Vibrio salmonicida *carries a large 170 MDa plasmid (pRSV1) harbouring an integron [22]. The integrase of the pRSV1 integron is clearly a vibrio-type integrase, not one of the class 1 or SXT constin integron integrases previously found on vibrio plasmids. This is shown by its strongly supported clustering with vibrio-type integrases in phylogenetic analyses (Figure 1). One of the gene cassettes of this integron encodes a transposase and another a dihydrofolate reductase type I usually found in class 1 integrons. It therefore seems that the *V. salmonicida *integron originates from a translocation (possibly transposition) event of a chromosomal vibrio-type integron to a plasmid and later acquired cassettes from class 1 integrons.

The frequent association of vibrio integrons with transposases, along with the variability in their genetic context and the DNA molecule carrying them (plasmid, large or small chromosome), suggest that some of them could be mobilized. As discussed above, there is strong evidence for intragenomic and intracellular translocation of integrons, but what remains to be established is whether lateral transfer of whole gene cassette arrays between strains and species can occur.

### Gene cassette transfers within and between *Vibrio *species

**Figure 5:**
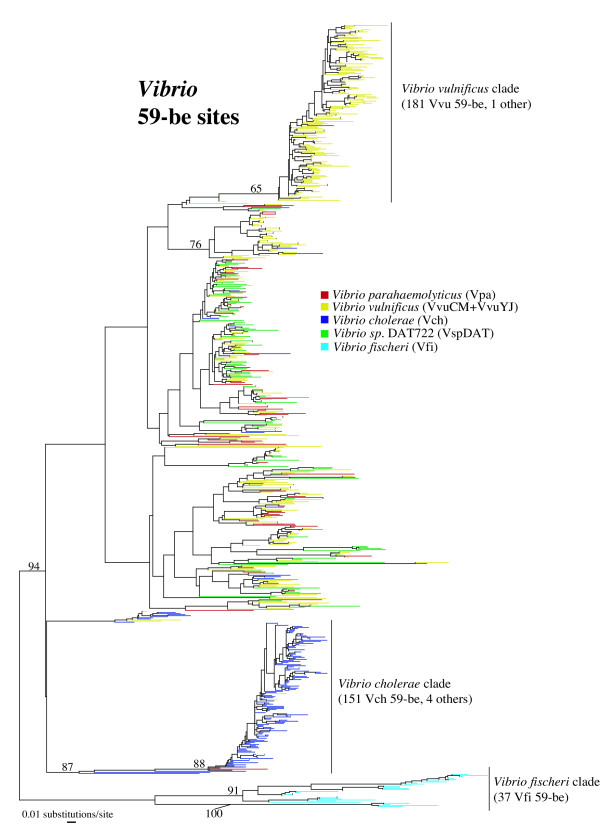
**Phylogenetic analysis of the 59-be sites found in *Vibrio *chromosomal integrons. **Each terminal branch is colour-coded according to the organism hosting the particular 59-be site it represents (see legend). The tree displayed in the best distance neighbor-joining tree obtained using PAUP*. Percent bootstrap support values represent the consensus of distance neighbor-joining trees obtained from 100 pseudo-replicates of the dataset. They are only displayed for important backbone nodes when the value exceeds 50%. 85 internal nodes are supported with >80% bootstrap value, 20 of which represent mixed species clades.

To examine the movement of gene cassettes between strains and species, we extracted and aligned all 59-be sites from the vibrio cassette arrays of *Vibrio sp*. DAT722, *V. parahaemolyticus *RIMD2210633, *V. cholerae *N16961, *V. vulnificus *YJ016, *V. vulnificus *CMCP6 and *V. fischeri* ES114(a total of 800 sequences). Figure 5 presents the phylogenetic analysis of 59-be based on this alignment. Three major clades emerge in the resulting tree: 1) All *V. fischeri *59-be sites cluster together to the exclusion of all others with strong statistical support (94% bootstrap value); 2) most (151/173 or 87%) *V. cholerae *59-be sites fall in a single clade along with four 59-be sites from other species with robust support (87% bootstrap value); 3) a large proportion (181/406 or 45%) of 59-be sites from either of the two *V. vulnificus *strains form a cluster with a single 59-be site from *V. parahaemolyticus *(weakly supported, 65% bootstrap value). The vast majority of clusters beside these three are formed of 59-be sites from a variety of species.

The major clades observed for *V. fischeri*, *V. cholerae *and *V. vulnificus *contrast with the absence of such phylogenetic structuring for *V. parahaemolyticus *or *Vibrio sp*. DAT722. The presence/absence of species-specific clades in the 59-be sites tree seems to be correlated with the phylogenetic distance of a species from others represented the dataset. In the most phylogenetically distant species of the dataset, *V. fischeri*, no exchange of 59-be sites with other species (indicated by mixed clades) is observed. *V. vulnificus *and *V. cholerae *also lack close relatives in the dataset, both being represented by relatively long branches in the *rpoB *tree (Figure 2). They both display one major species-specific clade along with some exchange of 59-be sites with other species. *Vibrio sp*. DAT722 and *Vibrio parahaemolyticus *are relatively close species (Figure 2) and neither of them displays a species-specific clade in the 59-be site tree.

Of 85 nodes with a bootstrap support value >80%, 65 contain only 59-be sites from the same species and 20 contain 59-be sites from multiple species, indicating transfer of gene cassettes between species. All species of vibrio are involved in such transfers except for the more phylogenetically distant *V. fischeri *(5 events involve *V. parahaemolyticus *as either donor or recipient, 8 *V. cholerae*, 8 *Vibrio sp*. DAT722 and 12 *V. vulnificus*). Among the 65 well-supported nodes containing 59-be from a single species only, 36 nodes contain 59-be sites solely from *V. vulnificus*. Of these 36, 30 contain 59-be sites from a single strain and 6 contain 59-be sites from both CMCP6 and YJ016. Transfer of gene cassettes can therefore be assumed to happen at both the subspecies and species levels among vibrios. The frequency of such transfer events seems to be broadly correlated with phylogenetic distance between the proponents (with a higher frequency between close relatives). Comparison of closely related vibrios, such as *Vibrio sp*. DAT722 and *V. parahaemolyticus *or individual strains of *V. vulnificus*, shows evidence of frequent transfer events that disrupt vertical inheritance patterns, ultimately leading to the absence of a major monophyletic clade for their 59-be sites. At the other extreme, transfer of cassettes between *V. fischeri *and other vibrios must be very rare or absent, as illustrated by the monophyly of all of its 59-be sites.

A phenomenon that could contribute to enhancing the degree of homogeneity between the 59-be sites found in a given species is gene conversion. This non-reciprocal recombination process occurs between relatively small tracts of DNA and is known to lead to the sequence homogenization of multiple paralogous genes found in a given organism, such as rRNA genes [21]. This could also be the case with 59-be sites, and linked with the process of nucleotide substitutions due to replication or repair errors, could lead to the creation and maintenance of distinct pools of 59-be sites in divergent organisms.

### Evolutionary rate of gene cassette arrays

Mixed phylogenetic clades composed of 59-be sites from different vibrio species, observed with *V. cholerae *and *V. metschnikovii *by Rowe-Magnus *et al*. [12] and here with a more substantial dataset, suggest frequent transfer of gene cassettes between vibrios. However, the phylogenetic structure present in the 59-be site tree, where 59-be sites from a given species cluster more frequently with others sharing a similar host, also suggest a correlation between phylogenetic distance and frequency of cassette exchange. This could be due to a lower efficiency of integrons at integrating gene cassettes from divergent species, as their 59-be sites did not co-evolve with the integrase and *attI *site of the host species. This hypothesis is supported by the recent findings of Biskri *et al*. [23] that the range of 59-be sites efficiently recombined by the *V. cholerae *integrase is narrower than the range of 59-be sites efficiently recombined by class 1 integrases. Performing similar assays of recombination efficiency using combinations of 59-be sites, *attI *sites and integrases from different vibrio species could allow direct testing of a correlation between phylogenetic distance and frequency of cassette exchange in vibrios.

Looking at the difference between two gene cassette arrays relative to their genomic context can yield insight on the rate of evolution of those arrays versus the rate at which the rest of the genome evolves. The two most closely related strains of *Vibrio *for which we have complete array sequences, *V. vulnificus *YJ016 and *V. vulnificus *CMCP6, share 30–39% of their gene cassettes (34 cassettes on an array size of 88 and 141 non-repeated cassettes, respectively). This represents a minimum of 107 gene cassette gain/loss events between the arrays (54 of YJ016 88 cassettes are not found in CMCP6 and the latter has 53 additional cassettes). This divergence is striking, as these strains are very closely related, displaying differences of only 6 bp in the 1500 bp of their *intI *genes and 7 bp in a 1000 bp fragment of their *rpoB *gene. Furthermore, the shared content of the array is also misleading for the estimation of cassette gain/loss events, as shared cassettes do not necessarily represent an evolutionarily conserved trait, but can have different histories, as is suggested by the lack of conservation in gene order between the arrays as opposed to the relatively conserved organization of the surrounding genomic genes [19]. Given that the impact of acquiring/losing a gene on organismal fitness would generally be greater than that of a nucleotide substitution, integrons are of immense evolutionary value for their host. Comparison of the cassette content of integrons from a large number of closely related strains, combined with a multi-locus sequence analysis to estimate the nucleotide substitution rate in the genome of these strains, could allow for a quantitative estimate of the relative evolutionary rate of integrons.

## Conclusion

The entire 82 kb integron gene cassette array of *Vibrio sp*. DAT722 was isolated and sequenced using a streamlined approach that could be used with most, if not all, integron-containing bacteria (Figure 1). The ease of preparation and high efficiency of fosmid libraries combined with the possibility to screen for integrons by PCR (targeting either of their main components, the integron integrase gene or 59-be sites), makes this approach widely applicable. Non-coding genetic elements such as 59-be sites are not readily identified by automated genome annotation tools, which focus mostly on identifying ORFs. Even for the identification of ORFs encoded within gene cassette arrays, these programs perform poorly, as they often identify the 59-be sites as being part of gene cassette ORFs or being ORFs themselves. This is in part due to the frequent use of alternative start codons in gene cassette encoded ORFs [24]. The approach implemented here, a simple motif search for the conserved ends of 59-be sites identifies gene cassettes with almost 100% efficiency, provided that sequence information is available from gene cassettes of an integron belonging to the same class as the target. By searching for the 59-be sites first and subsequently identifying ORFs that fall in-between these elements (allowing for minimal overlap), the accuracy of the annotation is improved significantly.

Although the mechanistic aspects of integrons are now relatively well understood (recombinase activity, preferential integration of cassettes at the *attI *site, etc.), little is known on their general biology, such as the size range of their gene cassette arrays, the composition and diversity of their genetic pool and their rate of evolution. Isolating and comparing gene cassette arrays from multiple closely related organisms is necessary to understand their role in natural environments. Because of the almost ubiquitous presence of large integron gene cassette arrays in vibrios and the wide distribution and ease of isolation of these bacteria, they represent an ideal model system to study this genetic element and its impact on natural populations. The comparative analyses performed here confirm previous studies as to the functional diversity contained in vibrio integrons as well as the high variability of their gene cassette content [12,19]. Additionally, evolutionary analyses of the gene cassettes of the *Vibrio sp*. DAT722 array revealed that these mobile elements can be recruited from genomic genes of various origins and can also be de-recruited, becoming a 'sedentary' gene after a non-specific integration event and the loss of its associated 59-be sites. This suggests a two-way interaction between the gene pool made up of gene cassettes and the larger gene pool composed of non-mobile bacterial genes. Our phylogenetic analysis of 59-be sites suggests that the gene cassette pool itself is fragmented along phylogenetic lines. The 59-be sites linked to gene cassettes are not homogeneous across all vibrios but tend to be more similar if found in closely related strains or species, suggesting more frequent transfers of gene cassettes among close relatives. Accumulation of sequence data on complete gene cassette arrays will allow for more precise estimates of the rates of gain, loss and transfer of gene cassettes and perhaps even on the degree of gene order conservation, provided that the arrays of very closely related organisms can be isolated.

## Methods

### DNA extraction and strain characterization

The *Vibrio harveyi *ATCC14126 reference strain was obtained from the American Type and Culture Collection. All other strains used in this study were isolated from marine environments in Australia: strains labeled DAT were obtained from aquaculture tanks used to raise mud-crab larvae in Darwin, Northern Territory (*Vibrio sp*. DAT722, *Vibrio sp*. DAT1706, *Vibrio sp*. DAT865, *Vibrio sp*. DAT868, *Vibrio sp*. DAT447); strains labelled SH originate from Sydney Harbour (New South Wales) seawater samples (*Vibrio sp*. SH32 and *Vibrio fischeri *SH36). All vibrios were isolated by plating water samples directly on thiosulfate citrate bile sucrose (TCBS) agar plates. Pure cultures of the strains were grown in rich medium (10 g tryptone, 10 g NaCl, 4.0 g MgCl_2_·6H_2_O, 1.0 g KCl, make up to 1 L with distilled H_2_O, adjust pH to 7.5 with 1 M NaOH). Genomic DNA was extracted as described in Wilson [25]. A 1127 bp DNA fragment of the RNA polymerase B gene (*rpoB*) (from nucleotide position 1315 to 2442 in reference to the *E. coli rpoB* gene), was amplified by PCR from the genomic DNA of all vibrio strains using general vibrio primers (rpoB1315F 5'-GGCGAAGTGGACGATATCGACC-3' and rpoB2442R 5'-GATCGAGTCTTCGAAGTTGTA-3'). A phylogenetic analysis of these *rpoB *fragments along with their homologs from completely sequenced vibrio genomes was performed to identify relationships between the isolates.

### PCR amplification and sequencing of PCR products

PCR amplifications were carried out in a final volume of 25 μl containing 1–5 ng of template DNA, 1.0 mM of each primer, and 12.5 μl of PCR Master Mix (PROMEGA). The reactions were performed with an initial denaturation at 94°C for 2 min., 30 cycles with a denaturation at 94°C for 30 sec., primer annealing at 55°C for 30 sec., and primer extension at 72°C for 1 min. PCR products were gel purified with the MinElute kit (QIAGEN) and cloned in TopoTA (INVITROGEN). Two clones were sequenced from both directions for each PCR product using an ABI377 automated DNA sequencer and BigDye v3.1 chemistry.

### Screening strains for the presence of integrons

Genomic DNA isolated from a pure culture of each strain was screened for the presence of an integron using one of two sets of primers: one set targeted part of *intI *(VintI_R 5'-CTGATATWMGWACMGTACAAGARC-3') and most of the gene known to be upstream of it in strains from the vibrio core group such as *V. parahaemolyticus*, VC1310 (VC1310_F 5'-CTGAATGTCTTATTTGCCTTTGG-3'); the second set targeted *Vibrio *gene cassettes 59-be sites (V59be_F 5'-CACACCTTARSNSRGCGTTA-3' and V59be_R 5'-TCCCTCTTGARCNSYTTGTTA-3'). Strains SH32 and SH36 were positive only for gene cassettes. Strains DAT722, DAT868, DAT865 were positive for both *intI *flanked by VC1310 and gene cassettes. Once the PCR products were confirmed by sequencing, these three strains were selected for genomic fosmid library construction.

### Construction and screening of fosmid libraries

Fosmid libraries were constructed from the genomic DNA of strains DAT722, DAT865 and DAT868 using the EPIFOS kit (EPICENTRE). Purified genomic DNA was run on a low-melt 1% agarose gel (AMRESCO) in a pulse-field gel electrophoresis apparatus (BIORAD) and the DNA of ~40 kb in size was cut out, purified from the agarose, ligated to the fosmid vector, packaged in phage capsids and used to infect *E. coli *as described in the EPIFOS kit manual. 480 colonies from each of the resulting libraries were picked from agar plates and used to inoculate 96-well blocks containing 1 ml of LB broth with 12.5 μg/ml of chloramphenicol (to select for the presence of fosmids) in each well. These cultures were grown overnight and glycerol stocks were made by mixing 140 μl of culture from each well with 60 μl of 50% glycerol in a 96-well plate. 10ul was sampled from each well and pooled by row in eppendorf tubes (120 μl per tube). The latter tubes were centrifuged to remove the LB broth and cells were resuspended in 15 μl of pH 7.0 Tris-HCl buffer. 1 μl of resuspended cells was added as template to PCR reaction tubes for screening. 40 PCRs were required to screen each library (1 reaction per 12 wells row, 8 rows per 96-well block, 5 96-well blocks per library). The PCR screening was done using primers targeting the *intI *gene and its upstream neighbor VC1310. For all positive rows of the blocks, 1 μl was taken out of each well from that row in the corresponding glycerol stock to inoculate a new PCR screening reaction. Clones testing positive in this second round of screening were re-streaked on LB agar plates containing 12.5 μg/ml of chloramphenicol, which were used to inoculate a liquid culture for extraction of pure fosmid DNA (as described in the EPIFOS kit manual). From 2 to 7 positive clones were obtained for each 480 clones library, with an average of 5 or ~1 % of positives.

Sequencing reactions were performed on one *intI */ VC1310 positive fosmid for each of the DAT722, DAT865 and DAT868 libraries using primer VintI_F (5'-CTTGTACGGTACGAATATCAGC-3') to obtain the complete sequence of their *intI *genes (completing the partial *intI *sequence on the *intI */ VC1310 fragment). An *intI*-bearing fosmid from the library with the most positive clones for *intI *(the DAT722 library, 7 positive clones / 480 total clones) was then selected for whole shotgun sequencing.

### Assembly of the complete *Vibrio sp*. DAT722 integron

The *intI*-containing DNA insert from the DAT722 library was cleaved from its fosmid vector using EcoRI and separated from it by PFGE. The insert DNA recovered from the low-melt agarose gel (EPIFOS kit manual) was then sheared in 1 kb fragments by nebullization and cloned in the TopoTA vector (INVITROGEN). Sub-clones were sequenced to obtain 8× coverage of the complete insert. Fragments were assembled using PHRED-PHRAP, which was also used to assess the quality of the sequencing. Low-quality regions were targeted by PCR giving a final average coverage of 11.8 (average for all the fosmid clones sequenced here). This yielded a 36 kb contig that included the *intI *gene, 33 kb of flanking DNA on one side and three gene cassettes integrated at *attI *on the other. To extend the array in the direction of the gene cassettes, a set of PCR primers targeting the last cassette of the contig was designed and used to re-screen the library. Both ends of library clones positive using this set of primers were sequenced to determine overlap with the original 36 kb contig. A fosmid clone with minimal overlap (2–5 kb) was selected for sequencing. This "walking" procedure was repeated until the end of the array was reached (i.e. gene cassettes were absent at one end of the last fosmid sequenced). This yielded a total of four fosmid clones of 36 kb, 32 kb, 30 kb and 37 kb in size, covering the entire 82 kb DAT722 integron gene cassette array as well as 33 kb of DNA on one side and 20 kb on the other (total length of 135 kb).

The integron gene cassette array DNA sequence determined in this study has been submitted to the GenBank nucleotide sequence database and assigned accession number [GenBank:DQ139261].

### Detection and annotation of gene cassettes in DNA sequence data

59-be sites were located in the DNA sequence using a Transact SQL procedure run in MSSQL 2000. The procedure searched for the conserved regions identified in the 59-be sites of previously analyzed vibrio strains (5': TAACAA, 3': GCGTTA). The results were then filtered by ensuring that the distance between the 5' conserved region and the 3' conserved region fell in a length range of 80 to 150 bp, as previously observed for other vibrio 59-be sites. These possible 59-be sites were then ranked based on how well they conformed to the consensus motif found in other vibrio 59-be sites on a scale of 1 to 20 and potential 59-be sites with scores below 15 (likely false positives) were discarded.

Once the 59-be sites had been identified, ORFs were located using another T-SQL procedure and then filtered by size (>50aa) and proximity to existing 59-be sites. ORFs that intruded too far (15 bp or more) into a 59-be site were disregarded or shortened. Overlapping ORFs were then examined manually and the most likely of them was retained (usually the ORF with a BLASTP match in the NCBI database). Once the 59-be sites, gene cassettes and ORFs had been found, they were automatically annotated in GenBank format using an ASP.net script. Cassettes in which no ORF was found according to the criteria defined above were used as a BLASTX query against the GenBank database to identify potential pseudogenes. Also, to confirm the "non-coding" nature of these cassettes with no apparent ORFs, all homologs of such cassettes found using BLASTN were aligned (>75% of potential non-coding cassettes had homologs). If all homologous cassettes were conserved across their whole length and no mutation pattern reminiscent of the variability of synonymous codon positions could be observed, the cassette was confirmed as non-coding. The SQL script used to annotate gene cassette arrays can be obtained from the authors upon request.

### Comparison of *Vibrio *gene cassette arrays

The DNA sequences of all integron gene cassette arrays found in completely sequenced vibrio genomes (along with the ORFs they encode) were retrieved from the KEGG database [26]. A PERL script was written to perform reciprocal BLASTP and BLASTN of the gene cassette contents of two arrays against each other (to detect homologs) and of an array against itself (to detect paralogs). The script can be obtained from the authors upon request. A cut-off e-value of 1E-10 was established to determine homology.

### Sequence alignments and phylogenetic analysis

The 800 59-be sites present in all gene cassette arrays from sequenced vibrio genomes as well as the DAT722 array were identified and retrieved using the Transact SQL procedure described above. They were then aligned using CLUSTALW [27]. The alignment was subsequently edited manually to remove ambiguous characters, leaving 123 unambiguous positions for phylogenetic analysis. The latter was performed in PAUP* 4.04b [28] and applying the Neighbor-Joining tree reconstruction method using the GTR (general time-reversible) nucleotide substitution model. Support values represent the consensus of 100 Neighbor-Joining trees constructed from bootstrap pseudo-replicates of the original dataset.

Phylogenetic analyses were also carried at the DNA level for the *rpoB *gene. Third codon positions did not display mutational saturation and were therefore included in the analyses. The *rpoB *trees were constructed with PAUP* 4.04b, applying the heuristic-search option and using the TBR branch-swapping algorithm. Maximum likelihood was used as the tree reconstruction method, with the nucleotide substitution model (GTR), gamma rates parameter α, proportion of invariable sites and nucleotide frequencies determined using MODELTEST [29]. The confidence of each node was determined by building a consensus tree of 100 maximum likelihood trees from bootstrap pseudo-replicates of the original dataset.

Amino acid alignments of the *Vibrio sp*. DAT722 gene cassette encoded ORFs and their homologs as well as the IntI protein family were constructed using CLUSTALW and edited manually to remove ambiguous characters. Maximum likelihood phylogenetic analyses were applied to these datasets using PROML with the JTT amino acid substitution matrix, a rate heterogeneity model with gamma-distributed rates over four categories with the α parameter estimated using TREE-PUZZLE, global rearrangements and randomized input order of sequences (10 jumbles). Bootstrap support values represent a consensus (obtained using CONSENSE) of 100 Fitch-Margoliash distance trees (obtained using PUZZLEBOOT and FITCH) from pseudo-replicates (obtained using SEQBOOT) of the original alignment. The settings of PUZZLEBOOT were the same as those used for PROML, except that no global rearrangements and randomized input order of sequences are available in this program. PROML, CONSENSE, FITCH and SEQBOOT are from the PHYLIP package version 3.6a [30]. TREE-PUZZLE and PUZZLEBLOOT can be obtained from the programs website [31].

## Authors' contributions

YB drafted the manuscript, conceived the study and performed the evolutionary analyses. CLN carried out the DNA sequencing and assembly of the fosmid clones and helped draft the manuscript. MJJ wrote the script for the annotation of gene cassette arrays. AR and BCM performed the functional annotation of gene cassettes and helped draft the manuscript. MRG participated in the conception and design of the approach, WFD took part in drafting the manuscript and helped coordinate the study. Finally, HWS participated in the experimental design and drafting of the manuscript.
